# Nightmare experience and family relationships in healthy volunteers and nightmare disorder patients

**DOI:** 10.1186/s12888-019-2299-9

**Published:** 2019-10-11

**Authors:** Chu Wang, Xu Shao, Yanli Jia, Chanchan Shen, Wei Wang

**Affiliations:** Department of Clinical Psychology and Psychiatry/ School of Public Health, Zhejiang University College of Medicine, Yuhangtang Road 866, Hangzhou, 310058 Zhejiang China

**Keywords:** Depressive state, Family relationship, Nightmare disorder

## Abstract

**Background:**

Nightmares are associated with parental bonding styles and various psychiatric disorders, but the exact connections between different nightmare experience features and family relationships in healthy volunteers and nightmare disorder patients are still unclear.

**Methods:**

We therefore invited 62 nightmare disorder patients and 135 healthy volunteers to undergo tests of the Nightmare Experience Questionnaire (NEQ), the Family Relationship Questionnaire (FRQ), and the Plutchik - van Praag Depression Inventory (PVP).

**Results:**

Besides the higher nightmare frequency and the higher PVP and four NEQ scale scores, the nightmare disorder patients had higher scores of FRQ Paternal Abuse, and lower ones of General Attachment, Maternal Encouragement, Maternal Freedom Release, and Paternal Freedom Release. The PVP was correlated with some NEQ and FRQ scales in both healthy volunteers and patients, and it functioned as a mediator between Physical Effect and Maternal Dominance in patients. Regarding predicting NEQ by FRQ, Paternal Abuse predicted Physical Effect, Maternal Dominance predicted Physical Effect and Horrible Stimulation, General Attachment predicted Horrible Stimulation (−) in healthy volunteers; Maternal Dominance predicted Physical Effect, Meaning Interpretation, and Horrible Stimulation, Paternal Freedom Release predicted Physical Effect (−), and Paternal Dominance predicted Meaning Interpretation and nightmare frequency in patients.

**Conclusions:**

Our study has demonstrated that the inappropriate family relationships were linked with different aspects of nightmare experience, especially in nightmare disorder patients.

## Background

Nightmares are extremely dysphoric and well-remembered dreams that usually involve efforts to avoid imminent dangers, which often result in awakening with rapid orientation and alertness [1]. Frequent nightmares of at least once per week, happen in about 5% of population [2–4]; while nightmare disorder, defined as repeated occurrences of nightmare and the consequent disturbance resulting in clinically significant distress or impairment in social, occupational, or other important areas of functioning [1], is prevalent in 37.3% mood disorders, 15.6% anxiety disorders, 66.7% posttraumatic stress disorder, and 31.1% personality disorders [5].

The exact etiopathology of nightmares is still unclear. Scholars have proposed several theories of the nightmare etiology, such as the psychoanalytic and the personality models, which emphasize that the modulation or transformation of emotion is a central function of dreaming, and the augmentation or disruption of this function is crucial to nightmares [6].

One of the more comprehensive models to date is the neurocognitive model [7], which highlights the roles of both affect load and affect distress in producing nightmares. Affect load is the combined influence of stressful and emotionally negative events on an individual’s capacity to regulate emotions, and nightmares occur when affect load is elevated by daytime emotional and memory demands. Affect distress is a long-standing disposition to experience heightened distress and negative affect in response to emotional stimuli, and this distress is correlated with prior abuse, neglect, insecure attachment, and trauma.

According to the neurocognitive model, many factors including family relationships which contributes to the psychological world of an individual, might influence the nightmare experience. Family relationships include parental bonding styles and attachment to family members, both of which play an important role in the psychological development and function of an individual [8, 9]. Previous results have shown, however, poor family relationships were linked to the increased affect load/ stress and emotional dysfunction of an individual; in consequence, they were more likely to develop the schema of vulnerability to harm [10], and to reduce the confidence dealing with difficulties and stress. The parental overprotection was related to child distress, such as to anxiety in children from normal family [11] and to depression in children suffering from cancer [12]. In clinics, the lack of parental support was significantly linked with the increased symptoms of posttraumatic stress disorder [13], the inappropriate rearing styles and less care or affection from parents were reported by the borderline personality disorder patients [14], and the higher maternal dominance and less paternal encouragement were correlated with more emotional disturbances in women with primary dysmenorrhea [15]. The reduced parental care and increased parental overprotection were linked to high level of neuroticism in adults [16], which in turn was significantly correlated with nightmare distress [17]. In addition, poor sleep quality was significantly increased among working aged adults with poor child-mother or child-father relationships [18], and the adverse childhood experience such as parental emotional abuse affected sleep of older adults [19]. However, up to present, there was no comprehensive picture of how different family relationships affect the frequency and different aspects of nightmare experience in the nightmare disorder patients, or in the healthy volunteers.

Scholars have developed several measures of family relationships and nightmares. For instance, the Nightmare Distress Questionnaire [20], Nightmare Emotions Scale [21], and Nightmare Behavior Questionnaire [22] were developed to measure the nightmare effect of distress, emotion, and daily behavior respectively; and the Parental Bonding Instrument [23], and the Egna Minnen av. Barndoms Uppfostran (My Memories of Upbringing) [24] were developed to measure the perceived parental bonding styles without measuring the family attachment. Recent literature shows that family relationships can be measured by the Family Relationship Questionnaire (FRQ) [25], which covers the following factors: General attachment, Encouragement, Abuse, Freedom release, and Dominance, on the other hand the measurement of nightmare experience, such as in the Nightmare Experience Questionnaire (NEQ) [26], covers Physical Effect, Negative Emotion, Meaning Interpretation, and Horrible Stimulation, making up for the deficiency of previous instruments.

Based on the previous studies and the neurocognitive model of nightmares, we have hypothesized that: (1) compared to healthy volunteers, the nightmare disorder patients score higher on nightmare frequency and nightmare experience domains, higher on the harsh and lower on the harmonious family relationship scales; and (2) the nightmare frequency and nightmare experience domains are positively associated with the harsh (i.e., abuse, dominance) and negatively with the harmonious family relationship scales (attachment, freedom release, encouragement), especially in nightmare disorder patients. We therefore invited a group of nightmare disorder patients, and a group of healthy volunteers who also had experienced idiopathic nightmares but between one to three times per year, to answer FRQ and NEQ. Since there was an association between the depressive symptoms and nightmares [27, 28], we also invited each participant to answer the Plutchik - van Praag Depression Inventory (PVP) [29], to measure their depressive mood.

## Methods

### Participants

One hundred and ninety seven participants raised from intact families were enrolled in this study. Sixty two patients with nightmare disorder (21 men, 41women; mean age: 25.56 years ±8.34 S.D.; age range 18–53 years) were recruited from a university hospital clinic. Some patients presented their major complaints of sleep problems such as the unrefreshing sleep, but later all patients were diagnosed as nightmare disorder by an experienced psychiatrist (WW) according to the DSM-5 [1]. One hundred and thirty-five healthy volunteers (50 men, 85 women, mean age 25.52 ± 7.40, age range 18–49) were recruited from university students or staff or community, who had experienced one to three times of nightmare per year. There was no significant difference between the two groups with respect to gender (χ^2^ (1, 195) = .19, *p* = .67), age (t (1, 195) = .04, *p* = .97), or education level (χ^2^ (5, 191) = 7.22, *p* = .21). All participants were confirmed to have no other confounding factors including schizophrenia, schizoaffective disorder, posttraumatic stress disorder, and had to be free from antipsychotic drugs or alcohol for at least 72 h prior to the test. Other exclusion criteria were: (a) having organic brain lesions according to the recent magnetic resonance imaging or computed tomography scans (for patients); (b) having prominent life events within the last 3 years; and (c) having watched horrible movies or videos within 72 h prior to the test. The study protocol was approved by a local ethics committee and all participants had given their written informed consents.

### Questionnaires

All participants were asked to complete the three questionnaires below in a quiet room, using a paper-and-pencil style.

A. The Nightmare Experience Questionnaire [26] has one item measuring nightmare frequency, and 20 items measuring the following four aspects (five items each): Physical Effect describing the adverse effects of physical health, appetite, and other daily activities after nightmares; Negative Emotion denoting the frightened, scared, or helpless emotions during nightmares; Meaning Interpretation describing an effort to interpret the nightmares or to value the information they might convey; and Horrible Stimulation assessing the scenes of terrifying activity or adventure, violence, or terror incidence in nightmares. Each item is rated using a five-point Likert rating scale (1 - very unlike me, 2 - moderately unlike me, 3 - somewhat like and unlike me, 4 - moderately like me, 5 - very like me). The internal alphas of these scales in the current study and in a previous study [26] are listed in Table 1.

**Table 1:** Scale internal alphas (Alpha 1, current study; Alpha 2, previous study) and scores (means ± S.D.) of the Nightmare Experience Questionnaire (NEQ), Family Relationship Questionnaire (FRQ), and Plutchik - van Praag Depression Inventory (PVP), in nightmare disorder patients (Patients, *n* = 62) and healthy volunteers (Controls, *n* = 135)

Scale	Alpha 1	Alpha 2	Patients	Controls	95% Confidence Interval
NEQ					
Physical Effect	.80	.85	10.00 ± 3.90*	6.64 ± 2.28	2.30 ~ 4.42
Negative Emotion	.80	.80	19.13 ± 4.04*	17.25 ± 4.75	.50 ~ 3.25
Meaning Interpretation	.62	.65	16.50 ± 3.57*	13.53 ± 4.11	1.78 ~ 4.17
Horrible Stimulation	.68	.69	14.44 ± 4.14*	12.21 ± 4.26	.94 ~ 3.50
Annual Frequency			32.45 ± 27.78*	2.10 ± 0.80	23.29 ~ 37.40
FRQ					
General Attachment	.60	.64	16.60 ± 3.61*	17.78 ± 3.71	−2.29 ~ − .07
Maternal Encouragement	.70	.71	14.92 ± 4.04*	16.75 ± 4.07	−3.06 ~ − .60
Maternal Abuse	.79	.79	8.21 ± 3.45	7.97 ± 3.87	−.90 ~ 1.37
Maternal Freedom Release	.86	.84	17.39 ± 4.61*	18.87 ± 4.25	−2.80 ~ − .16
Maternal Dominance	.67	.65	12.05 ± 3.74	11.86 ± 3.38	−.87 ~ 1.25
Paternal Encouragement	.78	.74	12.26 ± 4.50	12.96 ± 4.67	−2.10 ~ .70
Paternal Abuse	.65	.77	8.47 ± 3.99*	7.24 ± 2.48	.13 ~ 2.32
Paternal Freedom Release	.86	.83	17.10 ± 4.82*	18.75 ± 4.37	−3.02 ~ − .28
Paternal Dominance	.61	.62	11.63 ± 3.38	11.47 ± 3.64	−.92 ~ 1.24
PVP	.85	.94	14.42 ± 8.05*	10.17 ± 6.30	1.95 ~ 6.55

Note: *p < .05 vs. healthy volunteers

B. The Family Relationship Questionnaire [25] measures the core features of the experienced family relationship up to 16 years old. It consists of 43 items including five items for measuring the general attachment; (for both parents) five items for encouragement, five abuse, five freedom release, and four dominance: General Attachment assessing the affectionate dependence of a child to family members and family environment; Encouragement assessing the parental love, care and support; Abuse describing the physical or spiritual maltreatment, or criticism from parents; Freedom Release describing the liberty of decision-making or daily behavior given by parents; and Dominance reflecting the machismo and patriarchal authority from parents. Participants were also asked to rate each item using a five-point Likert rating scale (as in above questionnaire). The internal alphas of these scales in the current study and in a previous study [25] are also listed in Table 1.

C. The Plutchik - van Praag Depression Inventory [29] consists of 34 items. Each item has three scale points (0, 1, and 2) corresponding to increasing depressive tendencies. Subjects have “possible depression” if they score between 20 and 25 or “depression” if they score above 25. The internal alphas of the inventory in the current study and in a previous study [30] are also listed in Table 1.

### Statistical analyses

Two-way ANOVA was applied to the mean scores of NEQ and FRQ scales in the two groups of participant. Once a significant main effect was detected, the Student *t* test was employed as a post-hoc comparison. The annual nightmare frequencies and the mean PVP scores in the two groups were evaluated by the Student *t* test. A *p* value less than .05 was considered to be significant for these group comparisons. The relationships between the PVP and FRQ/ NEQ scales were assessed by the Pearson correlation test. Multiple linear regression (stepwise method) was employed to search for the relationships between FRQ and NEQ scales, taking the FRQ ones as predictors. Meanwhile, taking the PVP as a mediator between FRQ and NEQ scales, the bootstrapping sampling procedure by SPSS version 19.0 (SPSS, Inc., Chicago, IL, USA) was used to assess indirect effects [31]. In order to avoid the chance of Type I error, we set the meaningful level of prediction (NEQ by FRQ) at the absolute beta greater than .25, and of correlation (PVP with FRQ or NEQ) at the *p* value less than .01.

## Results

Compared to healthy volunteers, nightmare disorder patients scored significantly higher on PVP (t (1, 195) = 3.67, *p* < .001); individual data showed that six patients scored between 26 to 30, and were considered as in depressive state. Patients experienced significantly more nightmares (t (1, 195) = 8.60, p < .001), and scored higher on NEQ scale scores (group effect, F(1, 195) = 47.63, mean square effect (MSE) = 1156.58, *p* < .001; scale effect, F (3, 585) = 227.55, MSE = 2888.47, *p* < .001; group × scale interaction effect, F (3, 585) = 1.55, MSE = 19.66, *p* = .20), with that patients scored significantly higher on all the four scales than healthy volunteers did (Table 1).

In addition, nightmare disorder patients and healthy volunteers scored significantly different on some FRQ scale scores (group effect, F(1, 195) = 5.29, MSE = 119.16, *p* < .05; scale effect, F (8, 1560) = 186.93, MSE = 2721.61, *p* < .001; group × scale interaction effect, F (8, 1560) = 3.27, MSE = 47.55, *p* < .01), with significantly higher scores on Paternal Abuse, and lower scores on General Attachment, Maternal Encouragement, Maternal Freedom Release, and Paternal Freedom Release in patients (also see Table 1).

In healthy volunteers, PVP was significant-positively correlated with Physical Effect (*n* = 135, r = .24, *p* < .01) and Maternal Abuse (r = .26, *p* < .01). In patients, PVP was also significant-positively correlated with Physical Effect (*n* = 62, r = .40, *p* < .01) and Maternal Dominance (r = .33, *p* < .01). No other significant correlations were found between PVP and FRQ or NEQ scales (all other *ps* > .01).

When considering the prediction of NEQ parameters by FRQ scales, the adjusted R^2^ were ranged from .04 to .14 in healthy volunteers, and from .05 to .20 in patients. In healthy volunteers, Paternal Abuse predicted Physical Effect; Maternal Dominance predicted Physical Effect and Horrible Stimulation; and General Attachment predicted Horrible Stimulation (−). In patients, Maternal Dominance predicted Physical Effect, Meaning Interpretation, and Horrible Stimulation; Paternal Freedom Release predicted Physical Effect (−); and Paternal Dominance predicted Meaning Interpretation and nightmare frequency (Table 2). In patients, PVP acted as a mediator between Maternal Dominance and Physical Effect (indirect effect = .12, *p* < .01, 95% Confidence Interval = .02 ~ .24). There was no other indirect effect found in patients or in healthy volunteers.

**Table 2:** Stepwise multiple linear regression predicting NEQ by FRQ scales in nightmare disorder patients (Patients, *n* = 62) and healthy volunteers (Controls, *n* = 135)

	Patients		Controls	
	Adjusted R^2^	β (B, standard error) predictors	Adjusted R^2^	β (B, standard error) predictors
Physical Effect	.11	.28 (.30, .13) Maternal Dominance^a^	.14	.31 (.21, .06) Maternal Dominance^a^
		−.26 (−.21, .10) Paternal Freedom Release^a^		.27 (.25, .07) Paternal Abuse^a^
Negative Emotion	--	--	--	--
Meaning Interpretation	.20	.48 (.46, .11) Maternal Dominance^a^	.04	.23 (.24, .09) Maternal Abuse
		.27 (.28, .13) Paternal Dominance^a^		
Horrible Stimulation	.08	.31 (.34, .14) Maternal Dominance^a^	.14	.26 (.32, .10) Maternal Dominance^a^
				−.29 (−.34, .10) General Attachment^a^
				.24 (.22, .08) Paternal Encouragement
Annual frequency	.05	.26 (2.11, 1.02) Paternal Dominance^a^	--	--

Notes: all *p*s < .05; ^a^ |β| > .25; dashes (--), no significant results

## Discussion

In accordance with our first hypothesis, we found that patients with nightmare disorder scored higher on PVP, lower on FRQ General Attachment, Maternal Encouragement, Maternal Freedom Release, and Paternal Freedom Release, but higher on Paternal Abuse, and higher on nightmare frequency and all four NEQ scales. The depressive mood was correlated with some NEQ and FRQ scales in both healthy volunteers and patients. In healthy volunteers, Physical Effect was associated with Maternal Dominance and Paternal Abuse, Horrible Stimulation with Maternal Dominance and General Attachment (−); in patients, Physical Effect with Maternal Dominance and Paternal Freedom Release (−), Meaning Interpretation with Maternal Dominance and Paternal Dominance, Horrible Stimulation with Maternal Dominance, and frequency with Paternal Dominance, which supported our second hypothesis. Thus, our findings that both positive and negative family relationships influenced nightmare experience support that the emotion, abuse, and attachment factors display their impacts on the nightmare affect load and affect distress as proposed in neurocognitive model [7], and the model-fitting was more pronounced in our nightmare disorder patients.

The higher PVP in patients was in accordance with previous results [32]. In both healthy volunteers and patients, the physical impairment after nightmare (Physical Effect) was correlated with PVP, which was in line with that the distress engendered by nightmare was significantly related to the depressive state in university students [33]. In healthy volunteers, the maltreatment perceived from mother (Maternal Abuse) was positively correlated with PVP, which accorded with that individuals with a history of childhood abuse were more likely to become depressed [34, 35]. In patients, PVP was positively correlated with the dominant control by mother (Maternal Dominance), which was consistent with that depression was associated with maternal overprotection [16, 36].

In our patients, the increased frequency and the elevated four NEQ scale scores were in line with previous results [37–39]. The higher maltreatment perceived from father (Paternal Abuse) agreed with that childhood abuse or maltreatment had a strong association with nightmares [40, 41]. Women reporting more severe forms of maltreatment also reported higher frequencies of disturbed dream [42]. Patients’ lower scores of General Attachment (or the emotional attachment to family), Maternal Encouragement (care and support from mother), Maternal Freedom Release (freedom obtained from mother), and Paternal Freedom Release (freedom obtained from father) were also supported by that the poor parental bonding [18, 19, 43] and insecure family attachment [44] were associated with nightmare and related distress.

We also found some associations between NEQ and FRQ scales in both groups of participant. In healthy volunteers, Paternal Abuse was associated with Physical Effect, which was in accordance with previous studies showing that childhood abuse affected nightmares greatly [40–42]. Maternal Dominance involves excessive and unnecessary maternal protection, intrusiveness, and infantilization, which might be related to adjustment difficulties of a child [36], and then to the increased level of distress [13]. The studies might support that Maternal Dominance was associated with Physical Effect and Horrible Stimulation (the terrifying activity or violence experienced in nightmares). Indeed, inappropriate parenting has a negative impact on emotional management strategies in children [45], which might influence the capacity to cope with nightmares and the distress caused by nightmares. Moreover, other results have shown that the secure family attachment contributed to the high sleep quality of adolescents [46], which might decrease the horrible experience in dreams. Therefore, the quality of interpersonal relationships, as measured by General Attachment, was negatively associated with Horrible Stimulation in healthy volunteers.

In patients, Paternal Freedom Release was negatively associated with Physical Effect. In fact, higher Paternal Freedom Release permitted children to express their willingness and contribute their own effort into daily practice [15]. Children with more freedom gained positive development, healthy promoting behaviour, and secure attachment, thus might have less physical complaints [9, 47]. Also in patients, the higher Maternal Dominance was linked with more Physical Effect, more Meaning Interpretation (the effort to interpret the nightmare), and more Horrible Stimulation. Similarly, higher dominant control by father (Paternal Dominance) was related to more Meaning Interpretation and more nightmare frequency. These associations might be explained by the following results. The neurotic participants reported more nightmare frequency [48], more negative aspects of nightmare such as terror symptoms [49], more physical complaints after waking, and more likely to seek the dream meaning [22]. It was also reported that less parental care and more parental overprotection were linked with higher level of neuroticism in adults [16, 50], which helped understand our results. Previous results have shown that depression was correlated with the distress engendered by nightmare [33], and with the maternal overprotection [16, 36], which helped explain the indirect effect of PVP between Physical Effect and Maternal Dominance in patients.

Nonetheless, FRQ Maternal Abuse, Maternal Dominance, Paternal Encouragement (care and support from father), and Paternal Dominance were not significantly different between nightmare disorder patients and healthy volunteers, which were not consistent with our first hypothesis. There were no FRQ scales associated with NEQ Negative Emotion (frightened, scared, or helpless emotions during nightmares) in both groups, implying that family relationships have no effect on the frightened, scared, or helpless emotions during nightmares. There were also no FRQ scales associated with nightmare frequency in healthy volunteers, implying that family relationships contribute less to the nightmare occurrence in normal conditions.

However, the present study suffers from several design flaws. First, although we have excluded the acute or posttraumatic stress disorders from our study, we failed to note the detailed life events in our participants. Second, although the recall of childhood experiences tended to be reliable and accurate [51, 52], both measures of family relationships and nightmare experience were still subject to recall bias. Third, we did not measure personality features, especially neuroticism, of our participants, since nightmare disorder might be related to either normal or disordered personality traits. Nevertheless, we have demonstrated that the inappropriate family relationships were associated with different aspects of nightmare experience, especially in patients, suggesting a good family relationship allows more senses of belonging and security and less emotional disturbance, not only in waking state but also in dreams, and suggesting that family dynamics during childhood may be relevant to nightmare disorders.

## Conclusions

We have found that nightmare disorder patients had higher scores of Paternal Abuse, and lower ones of General Attachment, Maternal Encouragement, Maternal Freedom Release, and Paternal Freedom Release than healthy volunteers did, and had more depressive tendency. We have also found that some factors of family relationship were associated with the nightmare experience, especially the dominance and abuse from parents. Our study thus supports the neurocognitive model of nightmare etiopathology on one hand, and provides implications for using family therapy in nightmare disorder on the other, as a means to decrease harsh and increase harmonious relationships within the family.

## Data Availability

The datasets used and/ or analyzed during the current study are available from the corresponding author on reasonable request.

## Abbreviations

FRQ: the Family Relationship Questionnaire
NEQ: the Nightmare Experience Questionnaire
PVP: and the Plutchik - van Praag Depression Inventory
